# Comparison between Radionuclide Ventriculography and Echocardiography for Quantification of Left Ventricular Systolic Function in Rats Exposed to Doxorubicin

**DOI:** 10.5935/abc.20160194

**Published:** 2017-01

**Authors:** Luciano Fonseca Lemos de Oliveira, João Lucas O'Connell, Eduardo Elias Vieira de Carvalho, Érica Carolina Campos Pulici, Minna Moreira Dias Romano, Benedito Carlos Maciel, Marcus Vinicius Simões

**Affiliations:** 1 Centro de Cardiologia - Faculdade de Medicina de Ribeirão Preto - Universidade de São Paulo (USP) - Brazil; 2 Universidade Federal de Uberlândia (UFU), MG - Brazil

**Keywords:** Radionuclide Ventriculography / methods, Echocardiography / methods, Ventricular Function, Left, Comparative Study, Rats, Doxorubicin

## Abstract

**Background:**

Radionuclide ventriculography (RV) is a validated method to evaluate the left ventricular systolic function (LVSF) in small rodents. However, no prior study has compared the results of RV with those obtained by other imaging methods in this context.

**Objectives:**

To compare the results of LVSF obtained by RV and echocardiography (ECHO) in an experimental model of cardiotoxicity due to doxorubicin (DXR) in rats.

**Methods:**

Adult male Wistar rats serving as controls (n = 7) or receiving DXR (n = 22) in accumulated doses of 8, 12, and 16 mg/kg were evaluated with ECHO performed with a Sonos 5500 Philips equipment (12-MHz transducer) and RV obtained with an Orbiter-Siemens gamma camera using a pinhole collimator with a 4-mm aperture. Histopathological quantification of myocardial fibrosis was performed after euthanasia.

**Results:**

The control animals showed comparable results in the LVSF analysis obtained with ECHO and RV (83.5 ± 5% and 82.8 ± 2.8%, respectively, p > 0.05). The animals that received DXR presented lower LVSF values when compared with controls (p < 0.05); however, the LVSF values obtained by RV (60.6 ± 12.5%) were lower than those obtained by ECHO (71.8 ± 10.1%, p = 0.0004) in this group. An analysis of the correlation between the LVSF and myocardial fibrosis showed a moderate correlation when the LVSF was assessed by ECHO (r = -0.69, p = 0.0002) and a stronger correlation when it was assessed by RV (r = -0.79, p < 0.0001). On multiple regression analysis, only RV correlated independently with myocardial fibrosis.

**Conclusion:**

RV is an alternative method to assess the left ventricular function in small rodents in vivo. When compared with ECHO, RV showed a better correlation with the degree of myocardial injury in a model of DXR-induced cardiotoxicity.

## Introduction

In recent decades, imaging methods assessing functional and structural cardiac parameters in small animals *in vivo* have been widely used to study the pathophysiological mechanisms of ventricular dysfunction in different models of cardiac disease, and to develop new therapies for heart failure (HF).^1-7^ These methods allow a longitudinal study of the animals, increasing the power of observation at lower costs.

Among measurable parameters, the left ventricular systolic function (LVSF) is a key variable to evaluate myocardial remodeling, degree of ventricular dysfunction, and prognosis of myocardial disease. Echocardiography (ECHO) has been widely used to assess the ventricular function in humans and models of cardiac disease, as it is a low-cost tool for rapid acquisition of images without requirement of radioactive isotopes.^1,2,8^ However, the echocardiographic evaluation, especially in small rodents, depends largely on the observer and has restricted interobserver reproducibility, limiting the detection of subtle changes.^9^

Radionuclide ventriculography (RV) is a technique often used in clinical practice with good accuracy and high reproducibility levels in serial evaluations for LVSF quantification.^10,11^ In addition, RV is considered by many as the gold-standard method to assess ventricular function, as it faithfully represents the volumes of the ventricular chambers at each moment of the cardiac cycle without assumptions of myocardial shape and/or geometry.^12-14^ However, few studies have demonstrated its application in models of cardiac diseases in small animals.^15,16^

Although the use of RV in models of experimental cardiac disease in small rodents has been described for quite some time,^15,16^ there have been no studies comparing results obtained with RV with those obtained by other imaging methods *in vivo*. The objective of this study was to conduct a comparative analysis of the ability of ECHO and RV in evaluating the global systolic performance of the left ventricle and correlate these results with the severity of cardiac structural changes detected by histopathological analysis in a model of anthracycline-induced cardiotoxicity.

## Methods

### Experimental animals

After approval by the Ethics Committee on Animal Experimentation of our institution, the study was performed in 29 male Wistar rats weighing approximately 250 g, obtained from the institution's animal room. The animals were kept in an air-conditioned room, with free access to water and standard chow, subjected to a rhythm of 12 hours of light/dark and controlled temperature. During all procedures, maximum care was taken to avoid inflicting unnecessary suffering to the animals.

### General Study Design

To achieve a broader observation of the accuracy of both imaging methods in variable ranges of LVSF impairment, we used different doses of intravenous infusion of doxorubicin (DXR). This approach also allowed us to obtain a wide dispersion of the investigated variables to correlate better the changes in cardiac function measured *in vivo* and the degree of *in vitro* histological lesions that served as the gold-standard method to assess myocardial injury.

Based on that, the animals received three different cumulative doses of DXR over 8 weeks: D-8 mg: total infusion of DXR 8 mg/kg administered as four weekly injections of 2 mg/kg (n = 8); D-12 mg: 12 mg/kg accumulated over six weekly injections of 2 mg/kg (n = 7); D-16 mg: 16 mg/kg administered as eight weekly injections of 2 mg/kg (n = 7). Seven control animals received injections of saline solution over 8 weeks.

All animals underwent noninvasive LVSF evaluation with *in vivo* imaging methods, ECHO, and RV at baseline and 2 weeks after the end of the period of infusion of the respective doses of DXR or saline.

### Medications administered

Adriablastina^®^ RD (doxorubicin hydrochloride, Pharmacia, Milan, Italy) was dissolved in saline solution (10 mg/5 mL) and infused intravenously. A solution containing ketamine hydrochloride (Vetbrands, Jacareí, São Paulo, Brazil; 20 mg/kg) and xylazine (Bayer, São Paulo, Brazil; 8 mg/kg) was administered by intramuscular injection for anesthetic induction before each intravenous injection of DXR and imaging evaluations. To euthanize the animals, we used an overdose of these anesthetics (40 and 16 mg/kg, respectively).

### Echocardiography

After sedation and trichotomy of the anterior chest region, the animals, while breathing spontaneously, were placed in the left lateral decubitus position and evaluated with ECHO Doppler with a two-dimensional, high-resolution ECHO system (Sonos 5500 Philips, Andover, MA, USA) and a sectorial transducer with a frequency of 12 MHz. The ventricular function was estimated by the calculation of the left ventricular fractional shortening, measured from the short-axis view of the left ventricle. The area shortening was determined by the formula: Delta Areas = (AD - AS)/AD x 100,^17^ in which AD and AS are the areas in the diastole and systole, respectively. The fractional area shortening has been shown to be effective in detecting left ventricular systolic dysfunction in experimental models of myocardial infarction in rats^18-20^ and has the advantage of considering the two-dimensional image of the short axis of the left ventricle, compared with the ventricular shortening fraction (ΔD%), which takes into account only a linear ventricular dimension in the diastole and systole.

The images obtained were recorded and archived for later off-line analysis by an observer blinded to the group to which the animals were allocated. All measurements were obtained by the same investigator and revised by another; both were experienced in obtaining and analyzing ECHOs in small animals.

### Radionuclide ventriculography

**Figure 1:**
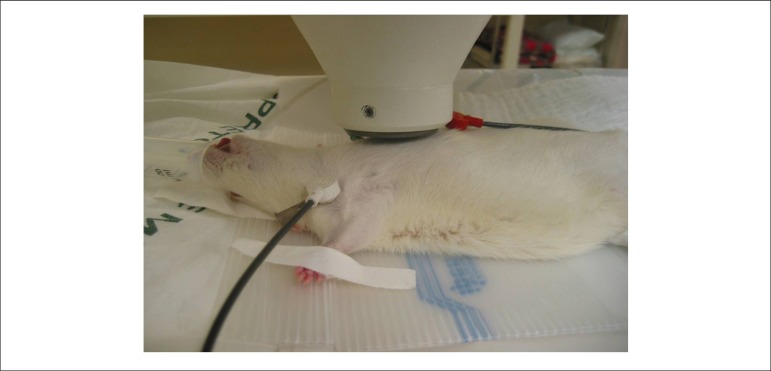
Positioning of the animal and the electrodes for radionuclide ventriculography.

After anesthesia, 75 *µ*g of stannous agent was injected into the tail vein. After an interval of 15 min, the animals received a new tail vein injection of 15 mCi of technetium pertechnetate-99m. Immediately after the administration of Tc-99m, the animals were taken to the gamma camera and positioned in a dorsal decubitus position under the detector. Three electrodes were implanted in the animals' hypodermis for electrocardiographic monitoring, positioned in the two anterior limbs and on the upper portion of the abdomen, as shown in Figure 1.

RV was performed with the gamma camera Orbiter-Siemens (Siemens, Erlangen, Germany), equipped with a pinhole collimator with a 4-mm aperture. Images were obtained in the left anterior oblique projection in a word format, 64 x 64 pixels matrices, synchronized with the electrocardiogram (ECG) with an acceptance window of 20% around the mean QRS duration value and with 32 frames per cardiac cycle. We acquired 200,000 counts per frame. The symmetric energy window of 20% focused on the Tc-99m energy photopeak was 140 keV.

**Figure 2:**
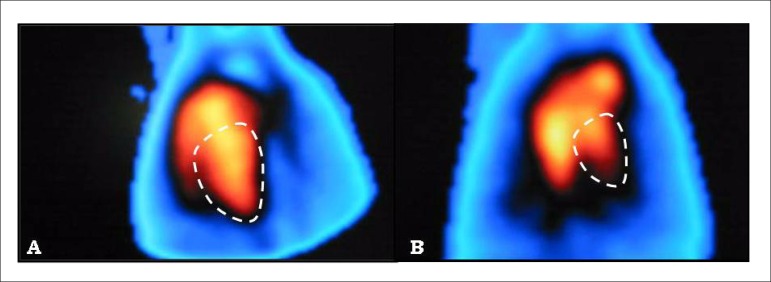
Images of the cardiac blood compartment labeled with ^99m^Tc in the left anterior oblique projection, in diastolic (A) and systolic (B) frames, allowing quantification of the LVSF after regions of interest were traced. LVSF = 76%.

To process the images, we used a commercially available software (planar gated blood pool, SMV America) in a dedicated workstation (NXT-P, Sopha Medical Vision). After semiautomatic detection of the edges of the left ventricle and with the help of parametric images of phase and count amplitude variation, a time *versus* activity curve was generated. From this curve, we calculated the LVSF, expressed as percentage (%), defined as the difference between the values corrected for the background radiation of the end diastolic and systolic counts divided by the value of the end diastolic count (Figure 2).

### Histology

After the animals had been euthanized, we quantified the extent of the myocardial fibrosis by measuring the collagen area in the myocardium. The hearts were sliced transversely, embedded in paraffin, and stained with picrosirius red. To quantify the collagen, we used the Leica QWin Software V 3.2.0 (Leica Imaging Systems Ltd., Cambridge, England) along with an optical microscope Leica DMR (Leica Microsystems Wetzlar GmbH, Wetzlar, Germany), a video camera (Leica DC300F, Leica Microsystems AG Heerbrugg, Switzerland), and an online computer. The values corresponding to the areas of fibrosis were obtained in relation to the total area of the left and right ventricles and septum, expressed as percentages. We evaluated 10 fields in the free left ventricular wall.

### Statistical analysis

To calculate the sample size, we established a (two-tailed) confidence interval of 95% and test power of 90%, assuming a standard deviation of 5 and 3 percentage units of LVSF for ECHO and RV, respectively; these values were obtained from a pilot study in control animals. As a result, a sample size of 22 animals was deemed appropriate to detect a difference of 5 ejective units between the methods. The sample size was calculated with a tool available online at www.openepi.com.

The results are expressed as mean and standard deviation. The Gaussian distribution of the variables was assessed with the Kolmogorov-Smirnov test. For comparison between mean LVSF values evaluated by the methods, we used paired Student's *t* test. To compare the mean values of the extension of the myocardial fibrosis between the control animals and the animals that received DXR, we used non-paired Student's *t* test. To analyze the correlation of the LVSF obtained by the imaging methods, we used the linear regression test and the least squares correlation. The Bland-Altman plot method was used for further analysis of the agreement between the LVSF measurements obtained by the two evaluated methods. Linear regression was also used to evaluate the correlation between the LVSF and the area of fibrosis.

All analyses were performed using the software GraphPad InStat, version 3.05, with a significance level of 5% (p < 0.05, two-tailed) for differences.

## Results

### Assessment of the left ventricular systolic function

**Figure 3:**
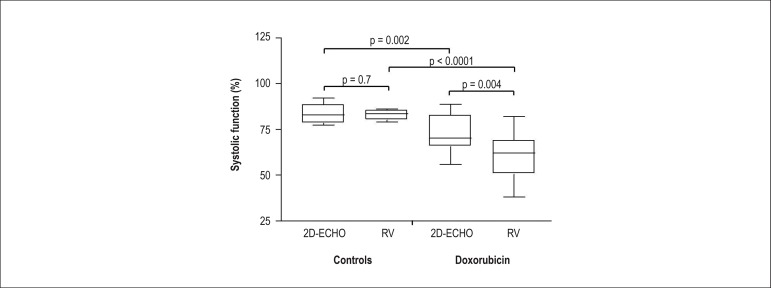
Graph showing the left ventricular systolic function obtained by two-dimensional echocardiography (2D-ECHO) and radionuclide ventriculography (RV) in animals in the control group and animals exposed to DXR.

Table 1 and Figure 3 summarize the results obtained.

**Table 1:** Summary of the mean and standard deviation results obtained from the evaluation of the left ventricular systolic function by echocardiography and radionuclide ventriculography in animals in the control group and animals that received doxorubicin

	ECHO (%)	RV (%)	Fibrosis (%)
DXR (n=22)	71.8 ± 10.1	60.6 ± 12.5	8.7 ± 3.2
Control (n=7)	83.5 ± 5	82.8 ± 2.8	2.3 ± 1
p	0.002	<0.0001	<0.0001

ECHO: echocardiography; RV: radionuclide ventriculography, DXR: doxorubicin.

The analysis of the LVSF in control animals showed values comparable to those obtained with RV and two-dimensional ECHO (82.8 ± 2.8% *versus* 83.5 ± 5%, p > 0.05).

A comparison between the control animals and the animals that received DXR indicated that both imaging methods showed lower LVSF values in the group receiving DXR (p < 0.005). These animals also showed a greater area of fibrosis when compared with the control animals (8.7 ± 3.2% *versus* 2.3 ± 1%, respectively, p < 0.05).

Additionally, the animals that received DXR exhibited significantly lower LVSF values assessed by RV (60.6 ± 12.5%) when compared with those obtained with ECHO (71.8 ± 10.1%, p < 0.05).

### Analysis of correlation and agreement

**Figure 4:**
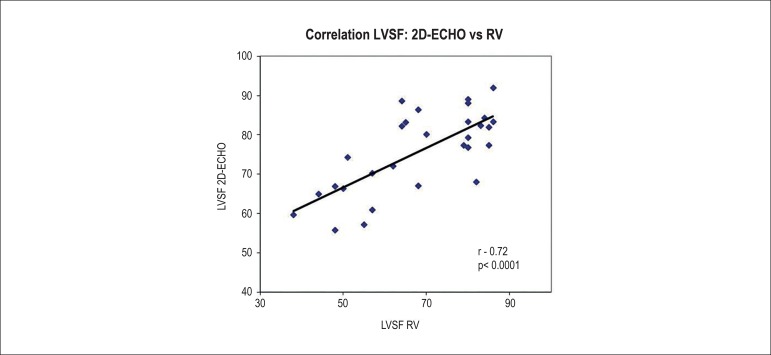
Graph correlating the left ventricular systolic function (LVSF) assessed by two-dimensional echocardiography (2D-ECHO) and radionuclide ventriculography (RV) in animals receiving different doses of doxorubicin (r = 0.72, p < 0.0001). (*Statistical test performed: linear regression analysis and Pearson's correlation coefficient).

Considering the entire study sample (including controls and animals exposed to DXR), individual LVSF measurements obtained by RV showed a significant and moderate positive correlation with those obtained by ECHO (r = 0.72, p < 0.0001) (Figure 4).

**Figure 5:**
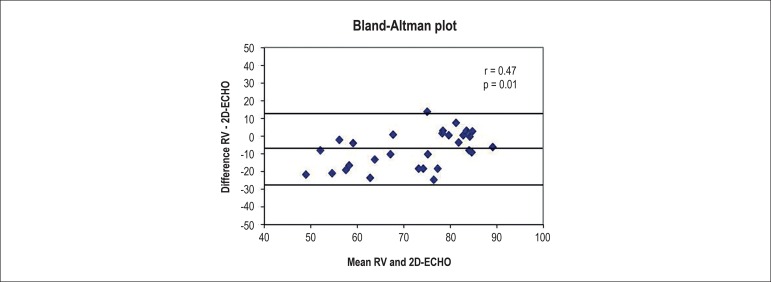
Bland-Altman plot indicating poor agreement between the imaging methods, showing lower values observed by radionuclide ventriculography (RV) in animals with decreased left ventricular systolic function.

A Bland-Altman plot analysis of the agreement between measurements (Figure 5) showed a mean difference (RV - ECHO) of -7.6 ± 10.3%, with limits of agreement of -28.1% to 12.9%. Analyzing the dispersion plot, we observed a significant positive correlation (r = 0.47, p = 0.01) between the mean values of the measurements obtained by RV and ECHO plotted against the difference of these same measurements, indicating that the methods do not have a good agreement for different ranges of LVSF values. This result suggests that RV estimates lower LVSF values than those estimated by ECHO in animals with a more compromised global systolic function.

### Correlation between the methods of in vivo functional evaluation and histological analysis

**Figure 6:**
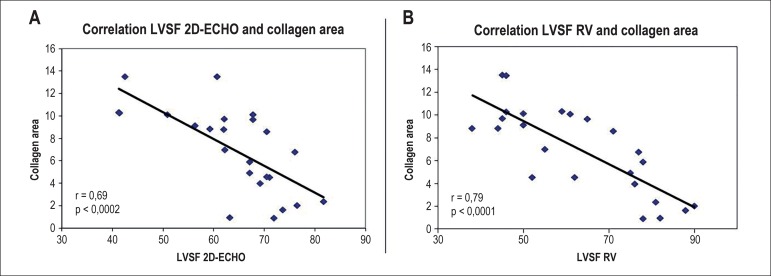
Graph correlating the mean left ventricular systolic function (LVSF) values evaluated by (A) two-dimensional echocardiography (2D-ECHO) and (B) radionuclide ventriculography (RV) and the percentage of accumulated collagen on histological analysis in animals receiving an infusion of 8, 12, and 16 mg/kg of doxorubicin.

After applying linear regression, we observed a moderate and significant negative correlation between the LVSF values measured by ECHO and the area of accumulated collagen on histology (r = -0.69, p = 0.0002). We found a stronger significant negative correlation between the LVSF assessed by RV and the area of accumulated collagen (r = -0.79, p < 0.0001) (Figure 6). Using multiple regression analysis, in which both imaging methods were included in the regression model, only the ventricular function measured by RV correlated independently with the percentage of accumulated collagen in the myocardium.

## Discussion

In many experimental models of cardiac disease in small animals, the serial assessment of the LVSF is the most widely used parameter to follow the changes in myocardial function due to disease progression or the impact of pharmacological intervention.^4^

In the present study using *in vivo* imaging methods, we evaluated the LVSF in control animals and in animals subjected to an experimental model of cardiotoxicity induced by DXR. This allowed us to compare two-dimensional ECHO and RV in the quantification of the LVSF in animals with different degrees of LVSF impairment, as well as to correlate these data with the quantification of the area of collagen tissue on histopathological analysis, which is considered a gold-standard method to assess the degree of myocardial injury in this cardiotoxicity model.

Our results showed that the LVSF values obtained by RV and ECHO were similar in control animals. However, in animals with severe ventricular dysfunction due to the cumulative infusion of DXR, the LVSF values obtained by RV were lower when compared with those obtained by ECHO.

Additionally, we observed a significant moderate Correlation between the LVSF results obtained by RV and ECHO. However, this type of analysis does not necessarily demonstrate the agreement between both methods.^21-23^ The Bland-Altman plot analysis indicated that despite showing a significant correlation in the regression analysis, the measurements were not in agreement in regards to different ranges of LVSF values. This analysis shows that, in addition to very wide agreement limits, ECHO evaluates relatively higher LVSF levels than does RV in animals with a more depressed systolic function.

Overall, these results indicate a greater sensitivity of RV when compared with ECHO in detecting left ventricular systolic dysfunction in this experimental model.

This interpretation of the data is reinforced by the results of the agreement analysis between the LVSF values obtained by each method and the extension of the collagen accumulation, which is an index of the degree of myocardial injury in this model. For this analysis, the RV results reached a stronger correlation than that obtained with ECHO, with the RV emerging as the only method displaying an independent correlation in the multiple regression model. This set of data reinforces the impression that RV provides a more accurate assessment of the LVSF.

Although no experimental studies have compared both imaging methods, prior clinical studies are consistent with our results when they showed that although ECHO and RV have a good general Correlation for LVSF measurement, both methods only have a moderate degree of agreement.^24-26^

In a study in patients after acute myocardial infarction (AMI), Ray et al. (1995) demonstrated a mean difference of -8 ± 10% in the LVSF evaluation between RV and ECHO, with limits of agreement of -28% to +12%,^27^ results that mirror to some degree those described in this study. Another study in patients after AMI has also shown that the LVSF was overestimated when evaluated by ECHO in comparison with RV, with wide limits of agreement.^28^ Bellenger et al.,^29^ while studying patients with stable HF, have shown a significant difference in LVSF evaluated by both methods. They also showed a moderate correlation (r = 0.44), but with wide limits of agreement between them, from -45% to 13%.

A low agreement between both methods of LVSF assessment has also been observed in patients with permanent pacemaker^30^ and after cardiac transplantation,^31^ studies in which ECHO overestimated the LVSF when compared with RV and cardiac magnetic resonance.

These differences between techniques, which have already been observed in the clinical setting, can be enhanced in the experimental scenario given the mechanical and geometrical differences of the myocardium in small animals. It should be noted that the contribution of the apical shortening in rodents can be different from that observed in humans, which would further compromise the geometric assumptions adopted in the ECHO for volume estimates.^32^

ECHO is the imaging method most widely used in large clinical trials and experimental studies, but it is dependent on geometric myocardial assumptions to estimate the LVSF. This becomes disadvantageous in several situations, as in the case of progressive dilation and consequent geometric changes of the left ventricle in HF.^33^ In this sense, biplane methods are considered more accurate than the M-mode method; however, they still extrapolate ventricular volumes measurements through geometric assumptions of the left ventricular cavity.^29^ In experimental animal models, especially rodents, another limiting factor of two-dimensional ECHO in quantifying the ventricular function is the loss of image quality in apical windows, generating poor endocardium definition and impairing the measurement of the left ventricular cavity shortening.^34-36^

It is plausible to assume that this limitation in accurately estimating the LVSF in dilated ventricular cavities with a more spherical conformation, concomitant to a more severe systolic dysfunction, is the main explanation for the results obtained in the present study. It is worth mentioning that to estimate the left ventricular ejection fraction, the RV assessment is based on the variation of counts directly proportional to the volume of blood in the ventricular cavity, and is a method, therefore, that is not significantly influenced by changes in the left ventricular cavity shape and geometry. In contrast, values obtained with two-dimensional ECHO are based on planar measurements, which are highly dependent on the geometric shape of the left ventricular cavity. Due to that, their accuracy changes in situations with more severe ventricular dysfunction in which the left ventricle takes a more spherical shape, in addition to presenting other deformations in cavity shape. The advent of three-dimensional ECHO and its availability in new equipment dedicated to small animals will probably bring substantial accuracy improvement in estimating the LVSF by ECHO, as it allows a direct measurement of the volumes of the ventricular cavities.^34-36^

It is important to remember that RV has several technical limitations, such as low spatial resolution, need for background correction, structure overlap, attenuation errors, and need for manipulation of ionizing radiation.^37^ In small rodents, limited spatial resolution is the greatest limitation of acquiring scintigraphic images with a gamma camera dedicated for clinical use. In fact, as the structures to be imaged in small rodents are 10 times smaller than the human organs, the spatial resolution of a conventional scintigraphic study (around 1.0 cm) should be proportionately increased to 1 mm. In our study, the use of a pinhole collimator with a 4-mm aperture was sufficient to considerably increase the spatial resolution of the image, with due visualization of the separation between the right and left ventricles (Figure 2) and maintenance of the 15-minute image acquisition, which is still suitable for an experimental study.

On the other hand, it is essential to emphasize that despite the high performance of RV in measuring the LVSF, this imaging method is more expensive and lacks information on other parameters of left ventricular remodeling such as diameter, wall thickness, and changes in the valvar apparatus. Therefore, we believe that RV, combined with ECHO, may become an additional tool for a thorough evaluation of models of cardiac diseases in small animals.

### Study limitations

Some limitations of this study should be highlighted. For example, we were unable to quantify the left ventricular ejection fraction by ECHO using the biplane Simpson's method due to a limitation in the resolution of the endocardium in the animals' apical images. It is possible that the use of a high-resolution ECHO equipment and a 30-MHz transducer dedicated to obtaining and analyzing images in small rodents could have yielded more reliable results. Similarly, a three-dimensional ECHO might have allowed the measurement of ventricular volumes and provided a far more accurate LVSF assessment. It is also noteworthy that our study did not include a gold-standard *in vivo* imaging method to measure the LVSF, such as cardiac magnetic resonance imaging.

## Conclusion

RV is an alternative method to evaluate the degree of left ventricular dysfunction *in vivo* in small rodents. When compared with ECHO, RV showed a better correlation with the degree of myocardial injury assessed by histopathology in a model of cardiotoxicity by DXR.

Our results suggest that although ECHO is a more available option, easy to use, and of low cost, RV may have in comparison a better performance, especially in sequential LVSF measurements in models of cardiac disease with changes in the left ventricular geometry.
